# Incomplete Kawasaki Disease Mimicking Deep Neck Infection: A Case With Tracheal Deviation and Retropharyngeal Edema

**DOI:** 10.7759/cureus.95923

**Published:** 2025-11-01

**Authors:** Kanako Ueda, Takateru Ihara, Yukari Atsumi, Tadamori Takahara

**Affiliations:** 1 Department of Pediatrics, Hyogo Prefectural Amagasaki General Medical Center, Amagasaki, JPN

**Keywords:** coronary artery aneurysm, deep neck infection, incomplete kawasaki disease, mucocutaneous lymph node syndrome, retropharyngeal abscesses, retropharyngeal edema, tracheal deviation

## Abstract

Kawasaki disease (KD) is usually diagnosed based on its characteristic mucocutaneous manifestations. However, in incomplete KD, where these features are not fully present, diagnosis is often delayed, and differentiation from other conditions, such as deep neck infection, is crucial. We report the case of a four-year-old boy who presented with anterior cervical swelling and lateral tracheal deviation, initially suspected to have a deep neck infection. On the fourth day of fever, he exhibited left anterior cervical swelling, trismus, and imaging findings of retropharyngeal edema. Despite antibiotic therapy for presumed deep neck infection, fever persisted, and echocardiography revealed coronary artery dilatation, raising suspicion of KD. Intravenous immunoglobulin was administered, but fever persisted, necessitating the addition of cyclosporine, which led to defervescence and improvement of inflammatory markers. Consequently, a coronary artery aneurysm developed, and incomplete KD was diagnosed. To our knowledge, KD presenting with tracheal deviation has not been previously reported. This case underscores the diagnostic and therapeutic challenges posed by KD presenting with clinical features mimicking deep neck infection.

## Introduction

Kawasaki disease (KD) is an acute febrile vasculitis of early childhood that predominantly affects medium-sized arteries and can involve the coronary arteries [1]. Such involvement may manifest as transient dilatation and, if untreated, progress to aneurysm formation. Most patients are younger than five years, with a peak around one to two years. KD is clinically diagnosed based on characteristic findings such as fever, rash, bilateral conjunctival injection, changes in the lips and oral cavity, changes in the extremities, and cervical lymphadenopathy. However, incomplete KD, defined as failure to meet the full clinical criteria even after serial assessment, is not uncommon and is associated with diagnostic delay and a higher risk of coronary artery abnormalities [2].

In some children, fever and cervical lymphadenopathy appear early, while other principal signs appear later. This “lymph node-first” presentation is frequently mistaken for bacterial cervical lymphadenitis or deep neck infection, which makes diagnosis challenging [3,4]. Cervical lymphadenopathy is the least frequent principal sign. In a registry-based cohort, it was observed in approximately one-quarter (24%) of KD cases [5]. In some cases, it can predominate and direct the initial differential toward infection [4,6,7].

Imaging provides supportive information but is not definitive. KD can produce retropharyngeal edema as a consequence of regional vasculitis with increased vascular permeability and interstitial edema; however, this finding is not specific and may also be seen in non-KD conditions [8,9]. On contrast-enhanced CT, ring enhancement, a hypodense fluid collection, intralesional gas, or a discrete drainable cavity more strongly support a bacterial abscess, whereas their absence favors non-suppurative processes such as KD-related edema [10].

To our knowledge, tracheal deviation caused by cervical swelling has not been previously described as a manifestation of KD. In clinical practice, it usually raises concern for space-occupying lesions such as an abscess or a tumor. We report an incomplete KD case in a four-year-old boy who presented with cervical swelling, retropharyngeal edema, and tracheal deviation that initially suggested deep neck infection.

## Case presentation

A four-year-old boy presented with a four-day history of fever and anterior cervical swelling first noted on day four of illness. His past medical history included hospitalization for respiratory syncytial virus bronchiolitis in infancy. Fever, decreased activity, and sore throat developed on day one of illness. Fever persisted from day one, peaking at 38.9°C on day two and remaining at or above 38.0°C thereafter. Rapid antigen tests for SARS-CoV-2, influenza, and adenovirus were negative. He remained able to take oral fluids and maintain the supine position. On day four, his guardian noted anterior cervical swelling, and repeat evaluation revealed a marked inflammatory response on blood testing, prompting referral to our hospital.

On admission, he assumed a sniffing position, with restricted neck movement and trismus. Diffuse swelling with erythema and tenderness was noted in the anterior to the left cervical region. Vital signs were stable: temperature of 37.1°C, heart rate of 132 beats/minute, respiratory rate of 36 breaths/minute, and SpO₂ of 99% (room air). No rash, conjunctival injection, oral mucosal changes, or extremity findings were observed.

**Figure 1:**
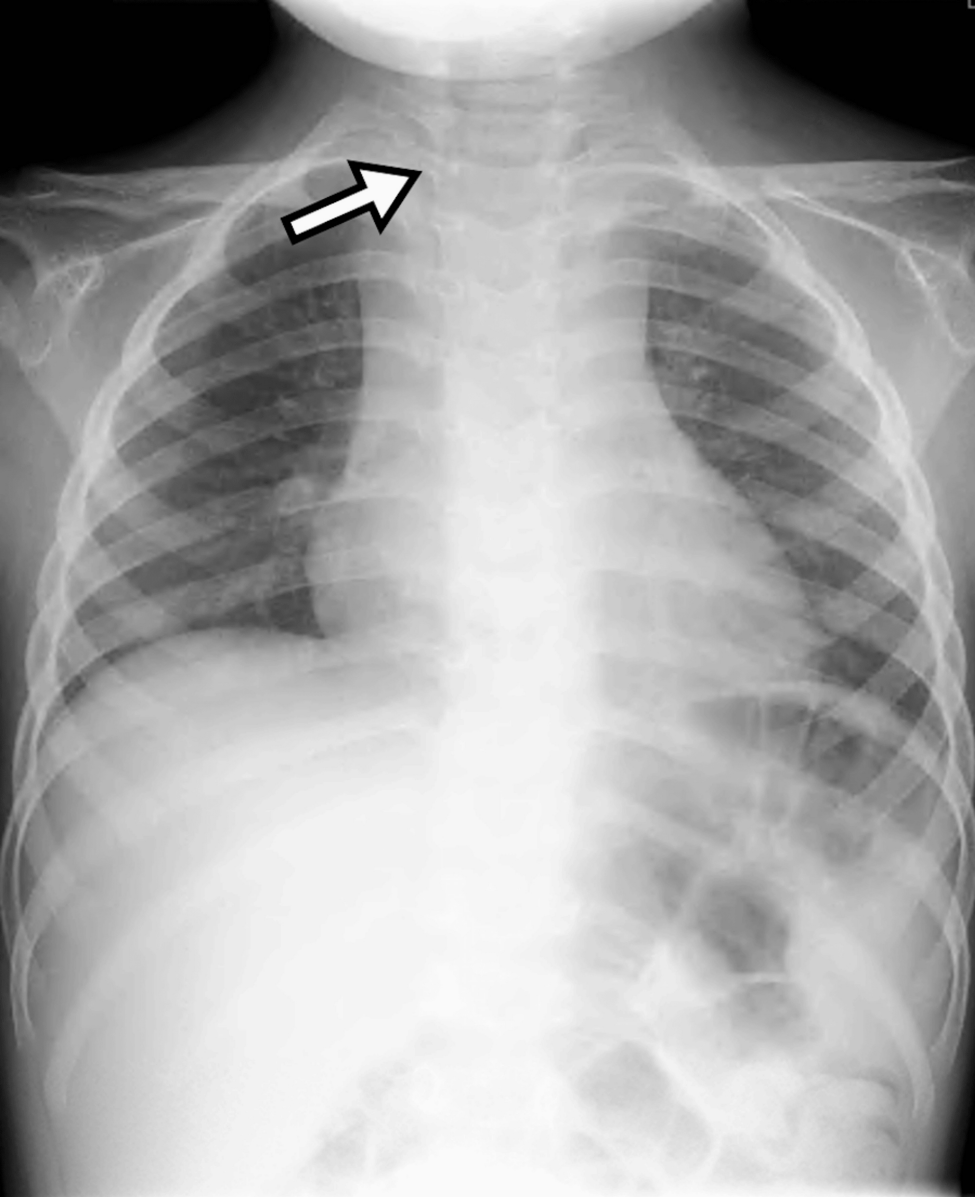
Chest radiography on the day of presentation. Chest radiography on the day of presentation showing rightward deviation of the trachea. The arrow indicates the displaced tracheal air column.

**Figure 2:**
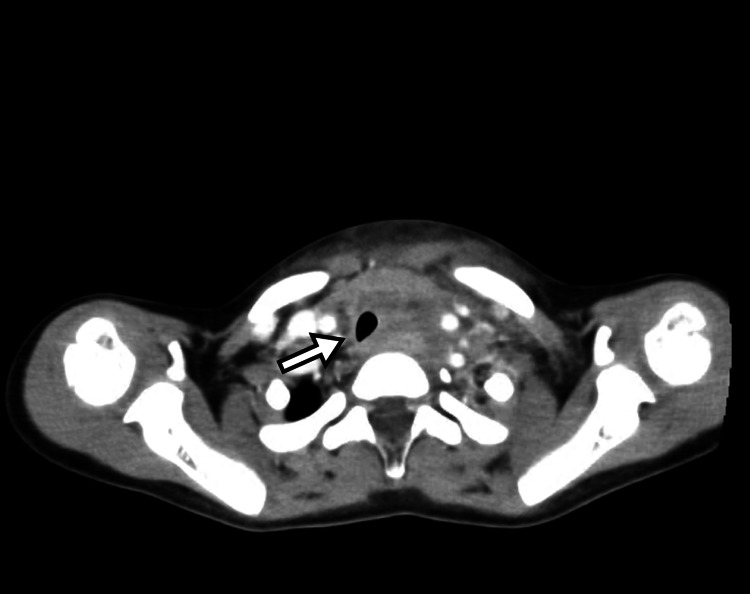
Contrast-enhanced CT on the day of presentation. Contrast-enhanced CT demonstrates displacement of the thyroid gland and trachea to the right, with diffuse soft-tissue swelling extending from the cervical region to the mediastinum. The arrow indicates the rightward-deviated trachea.

**Figure 3:**
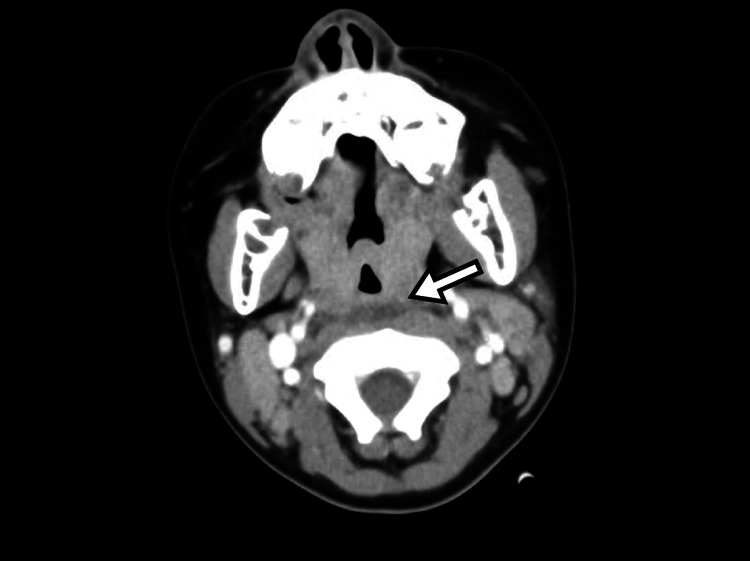
Retropharyngeal edema without abscess formation. Contrast-enhanced CT shows retropharyngeal edema without ring enhancement, low-attenuation fluid collection, intralesional gas, or any drainable collection. The arrow indicates the retropharyngeal edema.

Chest radiography revealed tracheal deviation to the right (Figure 1). Contrast-enhanced CT demonstrated displacement of the thyroid gland and trachea to the right, with diffuse soft tissue swelling extending from the cervical region to the mediastinum (Figure 2). Bilateral cervical and mediastinal lymphadenopathy and retropharyngeal edema were noted. No ring-enhancing collection, hypodense fluid collection, intralesional gas, or drainable collection was identified, findings that argue against abscess formation (Figure 3). Laboratory testing on admission showed leukocytosis with neutrophilia, thrombocytosis, elevated C-reactive protein (CRP) and procalcitonin, with mild decreases in albumin and sodium; total bilirubin and alanine aminotransferase were within reference ranges.

**Figure 4:**
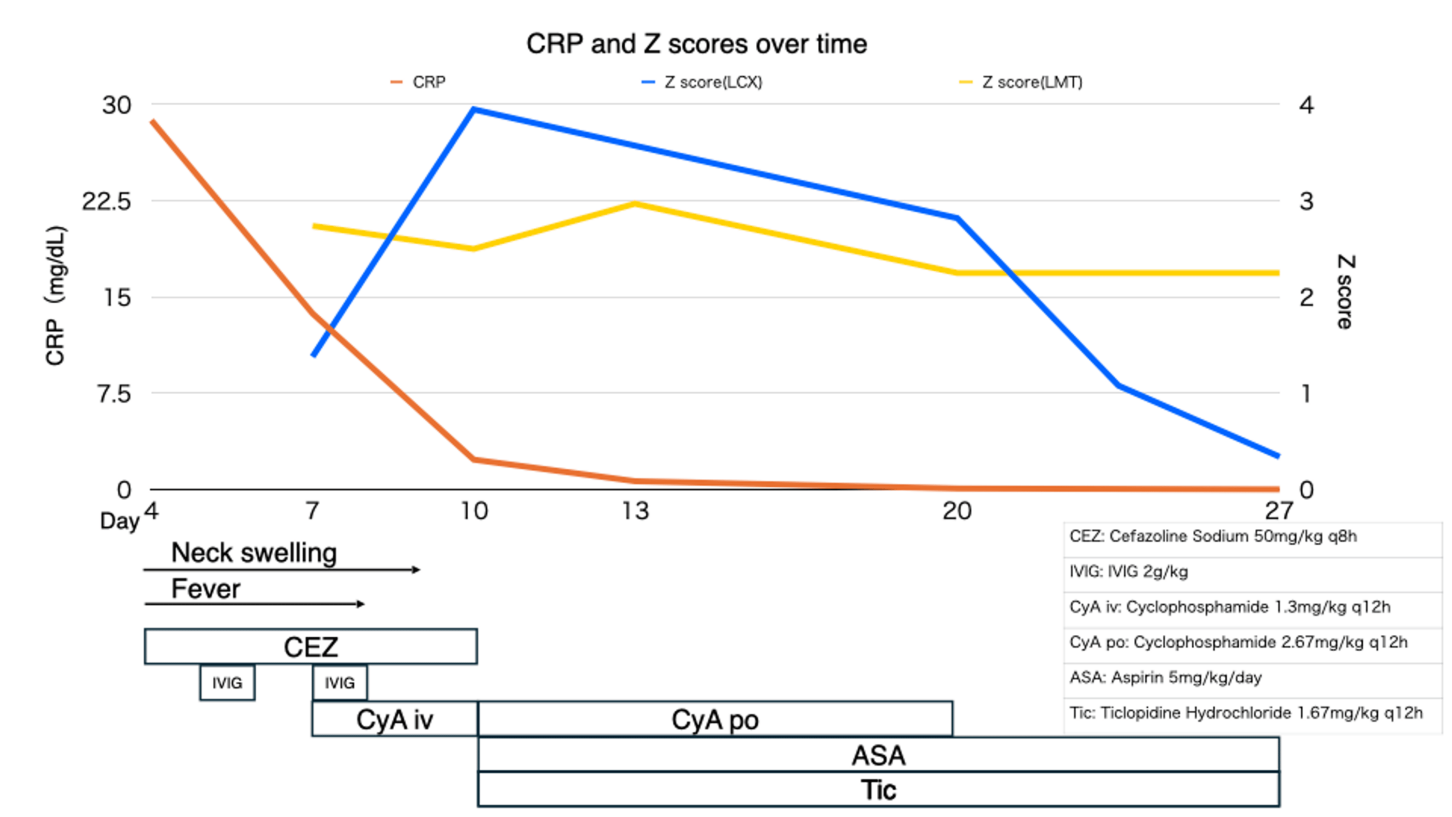
Timeline of clinical course with treatments, inflammatory markers, and coronary artery Z-scores. The figure illustrates the clinical course, including fever and cervical swelling, along with treatments (cefazolin, intravenous immunoglobulin (IVIG), cyclosporine, aspirin, and ticlopidine). Changes in C-reactive protein (CRP) levels and coronary artery Z-scores (left main trunk and left circumflex artery) are depicted over time.

Deep neck abscess was initially suspected, and intravenous cefazolin at 50 mg/kg every eight hours was started, along with symptomatic treatment. Before antibiotic initiation on the day of presentation, blood cultures were obtained and later showed no growth. On day five, although cervical mobility and general condition improved, fever persisted, and cervical swelling remained unchanged. Echocardiography revealed coronary artery dilatation. The subsequent changes in CRP levels, along with coronary artery Z-scores, are depicted in Figure 4. We evaluated the left main trunk, left anterior descending, left circumflex (LCx), and right coronary artery. Body surface area was calculated by the Mosteller formula, and Z-scores were computed using the Japanese pediatric Lambda-Mu-Sigma equations by Kobayashi et al. [11]. The maximum Z-score during hospitalization was 3.95 in the LCx (3.5 mm). Differential diagnoses, including juvenile idiopathic arthritis, collagen vascular diseases, and malignancy, were excluded, as hepatosplenomegaly, complement abnormalities, hyperferritinemia, or immunoglobulin elevation were absent. Because of the persistent fever and coronary findings despite antibiotic therapy, the patient was diagnosed with incomplete KD, and intravenous immunoglobulin (IVIG) at 2 g/kg was administered.

**Figure 5:**
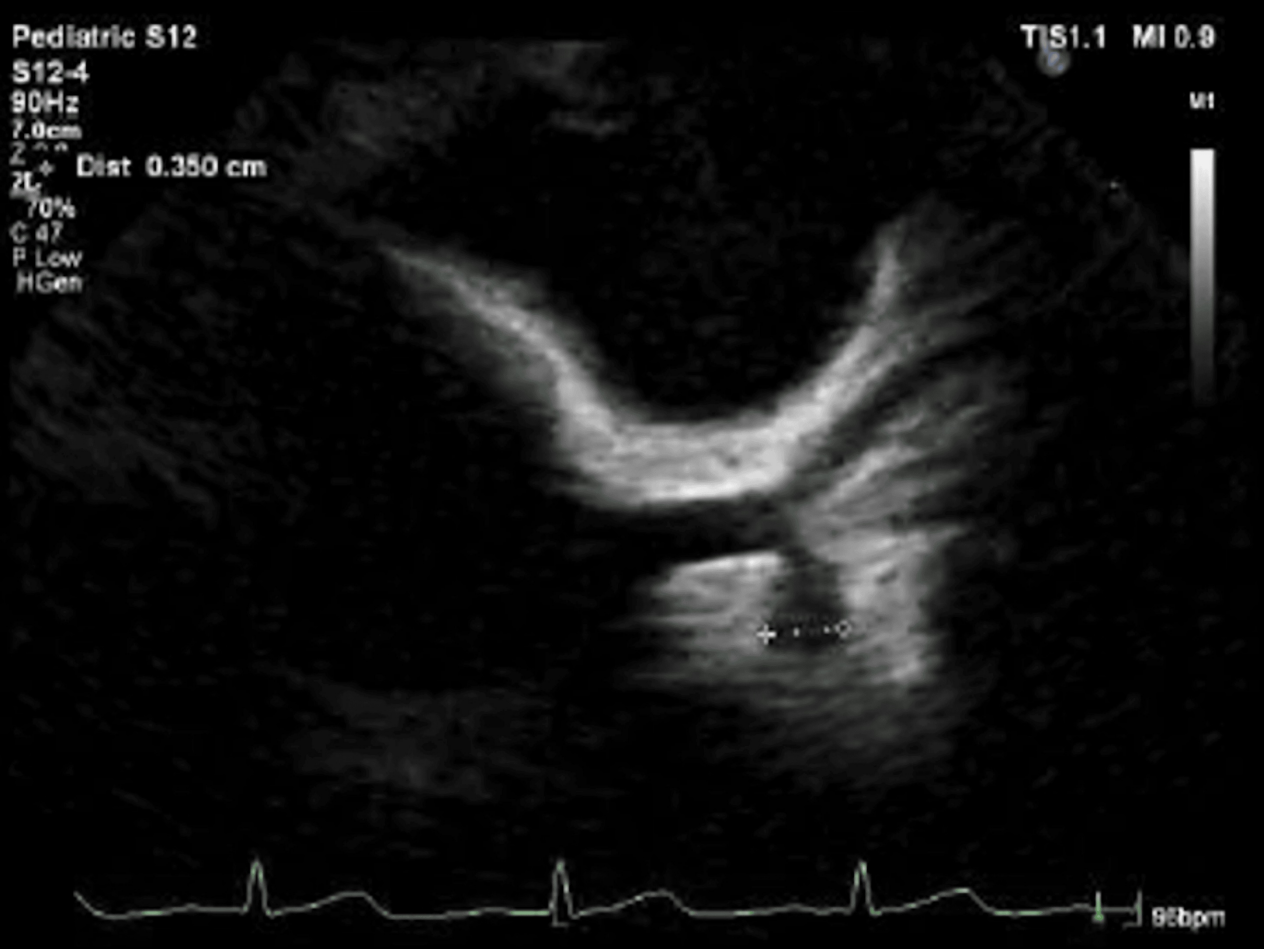
Transthoracic echocardiography on day 10 showing coronary involvement. Left circumflex coronary artery aneurysm (3.5 mm; maximum Z-score 3.95).

After the first dose of IVIG, his activity and oral intake improved, but his fever persisted at 38.0°C. On day seven, body temperature was 38.4°C, and echocardiography demonstrated progressive coronary artery dilatation (Figure 4), prompting initiation of second-line therapy with an additional dose of IVIG (2 g/kg) plus cyclosporine for a total of 14 days, administered at 1 mg/kg every 12 hours (2 mg/kg/day) for the first three days and then 2 mg/kg every 12 hours (4 mg/kg/day) thereafter. This approach was consistent with the 2020 revision of the Japanese Society of Pediatric Cardiology and Cardiac Surgery guideline, which permits cyclosporine as a second-line option in patients who did not receive cyclosporine in initial therapy (Class IIb, Level C) [12]. In accordance with our institutional protocol, glucocorticoids are preferred when no coronary involvement is present, whereas cyclosporine is selected when coronary artery changes are documented; therefore, cyclosporine was chosen in this case. By day eight, defervescence was achieved, and by day nine, the cervical swelling had completely resolved. On day 10, inflammatory markers had improved, yet echocardiography demonstrated a LCx coronary artery aneurysm (3.5 mm; maximum Z-score 3.95) (Figure 5). Dual antiplatelet therapy was initiated, and the patient was discharged on day 11.

**Figure 6:**
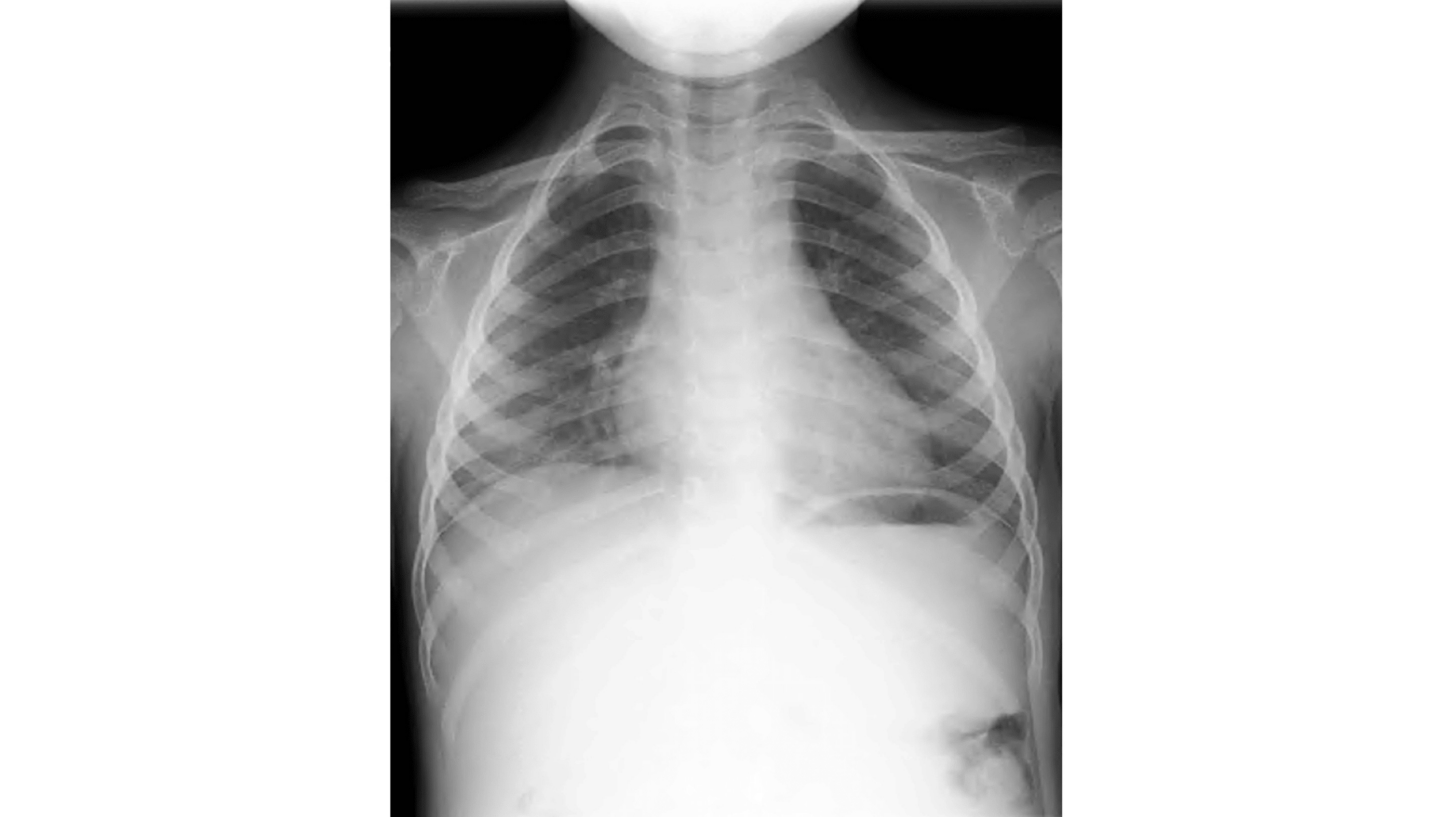
Chest radiography at one month after onset. Chest radiography confirming the resolution of tracheal deviation one month after disease onset.

As KD recurs in approximately 2% to 4% of patients, with most recurrences occurring within the first one to two years after the index episode [1,2], we scheduled follow-up visits at 2, 3, 6, and 12 months after onset. At the visit one month after onset, echocardiography showed resolution of the aneurysm with normalization of the coronary arteries, and chest radiography confirmed resolution of tracheal deviation (Figure 6). As of 22 months after discharge, there has been no recurrence of fever, cervical swelling, or other KD features. In addition, no clinical features suggestive of collagen vascular disease or autoinflammatory syndromes have appeared (for example, recurrent unexplained fevers, rash, arthritis, or serositis), and there have been no findings concerning for malignancy (for example, persistent or progressive lymphadenopathy, hepatosplenomegaly, weight loss, or night sweats). Serial echocardiography has shown sustained normalization of coronary dimensions, further supporting the final diagnosis of incomplete KD. Annual follow-up, with echocardiography as clinically indicated, is planned through five years after onset, in accordance with long-term follow-up recommendations from the Japanese Circulation Society and the Japanese Society of Pediatric Cardiology and Cardiac Surgery [13]. Written informed consent for publication of this case report and accompanying images was obtained from the patient’s guardian.

## Discussion

KD is an acute systemic vasculitis of childhood, with diagnosis based primarily on clinical findings [1]. Incomplete KD, where criteria are not fully met, may be diagnosed only after recognition of coronary artery lesions [2]. In this case, clinical presentation with anterior cervical swelling, sore throat, and persistent fever led to suspicion of deep neck infection. CT demonstrated retropharyngeal edema and soft tissue swelling, causing lateral displacement of the trachea. However, the absence of abscess formation, poor response to antibiotics, and emergence of coronary dilatation suggested KD.

Several reports describe Kawasaki disease mimicking deep neck infection. These cases showed retropharyngeal edema and soft tissue swelling, with KD recognized only after antibiotics failed and KD-suggestive findings appeared [4,6,7]. Kanegaye et al. also described “node-first presentation” of KD, in which cervical lymphadenopathy is the initial manifestation [4]. Katsumata et al. reported retropharyngeal edema in 82% of KD patients, but also in 30% of non-KD patients, highlighting limited diagnostic specificity [8]. Nomura et al. demonstrated that findings such as “ring enhancement” or “airway mass effect due to hypo-dense areas” are specific for bacterial abscess and not observed in KD [10].

In our case, retropharyngeal edema was present without ring enhancement or hypodense areas, distinguishing it from a typical bacterial abscess. Tracheal deviation is usually associated with mass lesions or abscesses; in this patient, it was attributed to diffuse inflammatory swelling. Failure to respond to antibiotics, together with progressive coronary dilatation, favored KD over bacterial infection. Thus, the final diagnosis of incomplete KD was made.

“Node-first presentation” of KD is more prevalent among older children (three years or older) and typically begins with fever and cervical lymphadenopathy before other principal signs, whereas classic KD most often presents at one to two years of age [14]. In this phenotype, inflammatory markers such as CRP tend to be elevated relative to other KD phenotypes, and several reports link this profile to a higher likelihood of resistance to IVIG and an increased risk of coronary artery involvement [15]. Procalcitonin, although classically elevated in bacterial infections, can also rise in acute KD; in lymph node-first presentations, values may overlap substantially with bacterial cervical adenitis, limiting its utility as a stand-alone discriminator [16]. Moreover, higher procalcitonin levels have been linked to treatment refractoriness (including IVIG resistance) and greater disease severity [17]. In light of these features, current guidance advises clinicians to consider KD even when only fever and cervical lymphadenopathy are present at first presentation, with other principal features appearing several days later. Taken together, these considerations indicate that lymph node-first KD is enriched among older children and carries greater risks of delayed diagnosis, treatment refractoriness, and coronary complications; therefore, when several days of antibiotic-unresponsive fever with cervical lymphadenopathy persist, KD should be strongly suspected, and early echocardiographic assessment should be pursued.

Importantly, despite IVIG administration, fever persisted and coronary dilatation progressed, indicating IVIG resistance. In KD, overall, initial resistance to IVIG occurs in approximately 10-20% of patients [1,2]. In presentations dominated by cervical lymphadenopathy (lymph node-first), evidence is mixed: some cohorts report rates similar to typical KD, whereas others suggest increased resistance among patients with prominent cervical lymphadenopathy [3,18]. Our patient’s persistent fever and progressive coronary changes are therefore consistent with IVIG-resistant KD. The addition of cyclosporine resulted in rapid defervescence, resolution of the inflammatory response, and regression of cervical swelling. Although a coronary aneurysm developed, regression occurred within one month, with no residual sequelae. This clinical course suggests an aggressive KD subtype with IVIG resistance, consistent with cyclosporine responsiveness and KD’s underlying immune-mediated pathophysiology [19]. The KAICA randomized trial evaluated adjunctive cyclosporine with first-line IVIG in patients predicted to be IVIG-resistant and showed improved coronary outcomes [20]. Because KAICA studied initial intensification, it does not directly inform second-line management after IVIG failure. In our case, cyclosporine was introduced together with the second IVIG dose in the setting of progressive coronary dilatation. This escalation followed the 2020 guideline of the Japanese Society of Pediatric Cardiology and Cardiac Surgery, which permits cyclosporine as a second-line option when it was not used in initial therapy (Class IIb, Level C), and is consistent with our institutional protocol that prefers steroids when no coronary involvement is present and cyclosporine when coronary changes are documented [12]. Alternative differential diagnoses, including collagen vascular disease, autoinflammatory syndromes, and malignancy, were systematically excluded, and the absence of recurrence during long-term follow-up further supported the diagnosis of incomplete KD.

## Conclusions

This case illustrates an incomplete presentation of KD with tracheal deviation. To our knowledge, reports of tracheal deviation in KD are rare. Recognition occurred after persistent fever despite antibiotics and later identification of coronary changes, rather than at the initial visit, which indicates diagnostic delay. The patient’s older age (four years) and elevated procalcitonin suggested bacterial infection and contributed to early misclassification, and tracheal deviation was misleading rather than diagnostic. The course included resistance to initial IVIG, escalation with a second dose and cyclosporine, a non-standard option in some guidelines but consistent with our institutional practice, and development of a coronary aneurysm despite treatment, followed by regression. As a limitation, incomplete laboratory documentation at presentation, including the absence of urinalysis, limits full diagnostic validation. Overall, the case underscores diagnostic difficulty and treatment challenges in KD with prominent cervical findings and supports maintaining suspicion for KD in antibiotic-unresponsive cervical swelling with retropharyngeal edema or airway deviation, together with serial reassessment and echocardiographic monitoring.
